# Emergence of Hypervirulent ST11-K64 *Klebsiella pneumoniae* Poses a Serious Clinical Threat in Older Patients

**DOI:** 10.3389/fpubh.2022.765624

**Published:** 2022-03-04

**Authors:** Tian Wei, Chengyun Zou, Jie Qin, Jianmin Tao, Li Yan, Jiangjun Wang, Hong Du, Fang Shen, Yanqin Zhao, Haiying Wang

**Affiliations:** ^1^Department of Clinical Laboratory, Yueyang Hospital of Integrated Traditional Chinese and Western Medicine, Shanghai University of Traditional Chinese Medicine, Shanghai, China; ^2^Department of Medical Technology, Yueyang Clinical Medical College, Shanghai University of Traditional Chinese Medicine, Shanghai, China; ^3^Department of Clinical Laboratory, The Second Affiliated Hospital of Soochow University, Suzhou, China; ^4^Department of Clinical Laboratory, Shanghai Fifth People's Hospital, Fudan University, Shanghai, China

**Keywords:** risk factors, multilocus sequence typing, hypervirulent, carbapenem-resistant *Klebsiella pneumoniae*, older patients

## Abstract

The carbapenem-resistant hypervirulent *Klebsiella pneumoniae* (CR-hvKP) poses a severe therapeutic challenge to global public health, and research on CR-hvKP in older patients remain limited. In this study, we aimed to investigate the clinical and molecular characteristics and risk factors of CR-hvKP infections in older patients. We retrospectively investigated older patients with carbapenem-resistant *Klebsiella pneumoniae* (CRKP) infections in the intensive care unit (ICU) between January 2020 and December 2020. The clinical data, and microbiological data including antimicrobial susceptibility testing, phenotype experiment and detection of carbapenemases, string test, virulence genes, capsular serotype-specific (cps) genes, and multilocus sequence typing, of the CR-hvKP group defined by the presence of any one of the virulence genes, including *rmpA, rmpA2, iucA, iroN*, and *peg-344* were compared with those of CR-non-hvKP strains. Of the 80 CRKP strains, 51 (63.8%) met the definition of CR-hvKP. The main mechanism of resistance to carbapenems was the presence of the *bla*_KPC−2_ gene. Sequence type (ST)11 (81.3%, 65/80) and ST15 (16.3%, 13/80) were the most common STs in CRKP strains. The minimum inhibitory concentration (MIC)_50_ values of the CR-hvKP group against the six tested antibiotics (ceftazidime, ceftazidime-avibactam, imipenem-avibactam, tigecycline, levofloxacin, and Cefoperazone-Sulbactam) exhibited elevated levels than the CR-non-hvKP group. Ceftazidime and imipenem by combining avibactam (4 μg/mL) significantly decreased the MIC_90_ values more than 16-fold than ceftazidime and imipenem alone against *Klebsiella pneumoniae* carbapenemase (KPC)-2-producing *K. pneumoniae*. Cardiovascular disease [odds ratio (OR) = 11.956] and ST11-K64 (OR = 8.385) appeared to be independent variables associated with CR-hvKP infection by multivariate analysis. In conclusion, higher MICs of the last line antibiotic agents (ceftazidime-avibactam, tigecycline) might be a critical consideration in the clinical management of older patients where the concentration of these toxic antibiotics matters because of underlying comorbidities. Caution regarding KPC-2-producing ST11-K64 CR-hvKP as being new significant “superbugs” is required as they are widespread, and infection control measures should be strengthened to curb further dissemination in nosocomial settings in China.

## Introduction

*Klebsiella pneumoniae* (*K. pneumoniae*), an important community-acquired and nosocomial pathogen, especially in the intensive care unit (ICU), causes widespread infections including pneumonia, bacteremia, urinary tract infection, endophthalmitis, liver abscesses, and sometimes even life-threatening septic shock. Two clinical pathotypes, hypervirulent *K. pneumoniae* (hvKP) and carbapenem-resistant *K. pneumoniae* (CRKP), often cause fatal infections (1). In most previous studies, the majority of hvKP identified to date are susceptible to most antimicrobials. However, the CR-hvKP strains are widely found, because the virulence genes and antibiotic resistance genes are transmitted by mobile genetic elements (2–4). In China, the prevalence of CR-hvKP infections is 0~25.8%, with large numbers of infections found in Henan and Shandong (5). The emergence of CR-hvKP could lead to the next clinical crisis, which has attracted worldwide attention (2).

In early studies, the definition of hvKP relied on clinical manifestations and phenotypes, which have proven to have poor specificity and sensitivity (6). Recently, except for *rmpA* and *rmpA2*, which have been associated with hypermucoviscous phenotype, multiple biomarkers of siderophore aerobactin (*iucA*), salmochelin (*iroN*) (4), and the putative metabolite transporter *peg344* on virulence plasmids (7) attributing to the hypervirulent phenotype of *K. pneumoniae* have been shown to have a high diagnostic accuracy for identifying hvKP (6). The emergence of CR-hvKP will be a great challenge for a clinician and will result in adverse outcomes. The growing number of older patients and patients undergoing surgery, transplantation, and chemotherapy will produce an even greater number of immunocompromised individuals at risk of these infections. So far, research on CR-hvKP in older patients has been extremely scarce (5). Therefore, a cognitive of the risk factors of infections, microbiological, and molecular characteristics in CR-hvKP will affiliate improved tracking and control of such CR-hvKP infection in older patients.

In response to this emerging problem, a retrospective study in older patients was conducted in a Chinese tertiary hospital to preferably understand the antimicrobial resistance, molecular, and clinical characteristics, risk factors, and epidemiology of CR-hvKP infections based on the newly recognized biomarkers and to guide the clinician to improve the monitoring, prevention, and treatment of CR-hvKP infection for older patients.

## Materials and Methods

### Bacterial Strains and Identification

We collected clinical CRKP strains from older patients between January 2020 and December 2020 from the ICU in Yueyang Hospital of Integrated Traditional Chinese and Western Medicine, Shanghai University of Traditional Chinese Medicine, which has a capacity of more than 1, 200 beds. The definition of older was the age of the patient being ≥65 years. All the strains were collected consecutively and non-duplicated, and only the first strain was included from the same patient. All the *K. pneumoniae* identification and the drug resistance of imipenem and ertapenem were performed using Vitek 2 system (bioMérieux, France), and further species identification was confirmed with MALDI-TOF MS system (Bruker Daltonics, Billerica, USA), and the resistance of meropenem was determined by a Kirby-Bauer disk diffusion susceptibility test. The CRKP was defined as an isolate with a minimum inhibitory concentration (MIC) of ≥ 2 μg/mL for ertapenem, ≥ 4 μg/mL for imipenem, or disk zone diameter ≤ 19 mm for meropenem in accordance with the breakpoints of 2020 Clinical and Laboratory Standards Institute (CLSI) guidelines (8). All the clinical information of patients with positive CRKP were collected from electronic medical records, including basic demographics, underlying diseases, clinical presentations, exposure to hospital care, treatment process, and outcomes.

### Antimicrobial Susceptibility Testing

The Antimicrobial Susceptibility Testing (AST) for the CRKP strains was further performed by the broth microdilution assay (BIO-NONT, China). The antibiotics included ceftazidime, ceftazidime-avibactam, cefepime, aztreonam, piperacillin-tazobactam, imipenem, imipenem-avibactam, meropenem, amikacin, polymyxin B, tigecycline, levofloxacin, and cefoperazone-sulbactam. The results were interpreted using 2020 CLSI guidelines (8), except for tigecycline and polymyxin B, which were interpreted according to the US Food and Drug Administration (FDA) standard (9) and the European Committee on AST (EUCAST) standard, respectively (10). The breakpoints of imipenem-avibactam and cefoperazone-sulbactam were referred to as imipenem and cefoperazone in CLSI, respectively (11, 12). The standard strains *E. coli* ATCC 25922 and *K. pneumoniae* ATCC700603 were used as quality control strains in each experiment.

### DNA Extraction

The strains were inoculated into the blood agar plate and incubated overnight at 35°C. The colonies were suspended in 0.5 ml sterile distilled water. The suspension was boiled at 100°C for 10 min, and centrifuged at 10, 000 rpm for 13 min. The supernatant was used as a template for DNA reaction. The DNA was stored at −35°C.

### Phenotype Experiment and Detection of Carbapenemases

Phenylboronic acid (PBA) and ethylenediamine tetra-acetic acid (EDTA) companying with imipenem were used to detect the phenotypes of *K. pneumoniae* carbapenemase (KPC) β-lactamase and metal β-lactamase (MBLs), respectively (13). In both tests, compared with imipenem alone, the diameter of the inhibition zone has increased ≥5 mm by PBA or EDTA, which is considered to be a positive combined-disc test result that shows the presence of KPC β-lactamase or MBL enzyme, respectively.

The presence of genes encoding carbapenemases (*bla*_KPC_, *bla*_NDM_, *bla*_IMP_, and *bla*_OXA−48_) were detected by PCR assay using the primers previously described (14, 15), which is listed in Supplementary Table 1. Then, the sequence analysis of PCR products of carbapenemase-encoding genes was then conducted (Sangon Biotech, Shanghai, China) and aligned in BLAST searches in the NCBI Genbank.

### Detection of Virulence-Associated Features and Capsular Serotype-Specific Genotyping

Hypermucoviscosity as a virulence factor was identified by the string test. If a viscous string longer than 5 mm is generated by stretching a bacterial colony on a blood agar plate with a bacteriology inoculation loop, the string test is positive (16). Five virulence genes including *rmpA, rmpA2, iucA, iroN*, and *peg-344* (6, 17, 18) and eight capsular serotype-specific (*cps*) genes (K1, K2, K5, K20, K47, K54, K57, and K64) (18–21) were identified by PCR as described previously. The primers are listed in Supplementary Table 1.

### Multilocus Sequence Typing (MLST)

Seven housekeeping genes (*gapA, mdh, phoE, tonB, infB, pgi*, and *rpoB*) were performed according to the protocol, which is described in online databases. The PCR amplified products were sequenced (Sangon Biotech, Shanghai, China), and the results were compared by the use of this website.

### Statistical Analysis

IBM statistical product and service solutions (SPSS) (version 19.0) was used for statistical analysis. The χ^2^ or Fisher's exact test was used for categorical variables. *P* < 0.05 was considered statistically significant. To further identify the variables associated with CR- hvKP infections, logistic regression was used. All the variables that revealed to be statistically significant in univariate analysis were included in the multivariate analysis.

### Ethical Approval

The study was approved by the research ethics committee of Yueyang Hospital of Integrated Traditional Chinese and Western Medicine, Shanghai University of Traditional Chinese Medicine. Informed consent was not needed due to the retrospective nature of the study.

## Results

### CRKP Strains and Virulence-Associated Features

A total of 80 consecutive cases of CRKP infection from ICU were included in this study. The percentages of 80 CRKP strains isolated from respiratory tract, urine, blood, pus, and puncture fluid were 72.5, 15.0, 6.3, 5.0, and 1.3%, respectively. CRKP strains were divided as CR-hvKP and CR-non-hvKP based on the presence of any one of the virulence genes, including *rmpA, rmpA2, iucA, iroN*, and *peg344*. The detection of the presence of virulence genes showed that 63.8% (51/ 80) strains were positive, suggesting to be carrying virulence plasmids, which were designated as CR-hvKP group, while the remaining 29 strains were designated as CR-non-hvKP group with negative results for virulence genes by PCR analysis.

**Figure 1:**
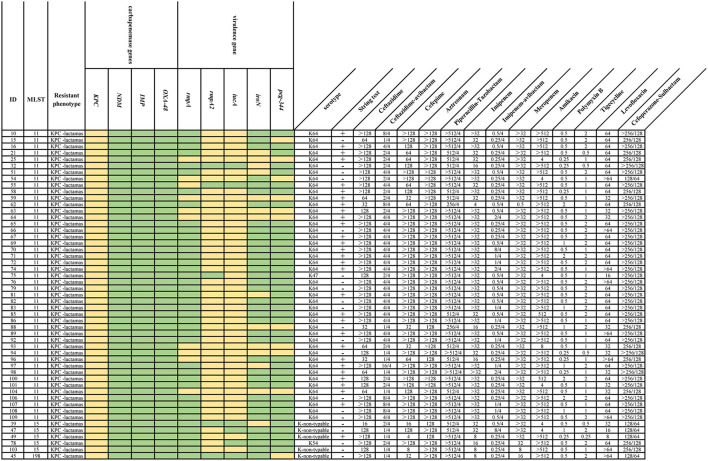
MLST, resistance, and virulence factors, and MIC values of 51 CR-hvKP strains. Multilocus sequence type (MLST), resistance, and virulence factors and minimum inhibitory concentrations (MICs) are shown. The presence of genes in a specific genome is represented by the light yellow box and the absence of genes is represented by a green box. Each row of the heatmap (middle) indicates a strain, and each column represents a gene that belongs to the indicated functional clusters shown at the top.

Five virulence-associated genes, *rmpA, rmpA2, iucA, iroN*, and *peg-344*, were detected in 51 strains (Figure 1), and the most common virulence gene was *rmpA2* (90.2%, 46/51), followed by *iucA* (88.2%, 45/51), *iroN* (27.5%, 14/51), *peg-344* (21.6%, 11/51), and *rmpA* (19.6%, 10/51). Seven (13.7%) strains had all five of the virulence genes, which indicates that these strains could probably carry the full length of the virulence plasmid pLVPK. Moreover, 41.2% (21/51) CR-hvKP strains showed negative results in the string test, whereas 17 CR-non-hvKP strains were positive, accounting for 58.6% (17/29).

For the eight serotypes closely related to hvKP, 65 (81.3%) strains belonged to K47, K54, or K64 serotypes. Additionally, there was no strain with K1, K2, K5, K20, or K57 serotypes in this study. Among all CRKP strains, capsular serotype K64 (77.5%, 62/80) was dominant. The frequency of K64 type (86.3 vs. 62.1%, *P* = 0.013) identified in CR-hvKP strains was significantly higher than CR-non-hvKP strains, whereas K-non-typable (34.5 vs. 9.8%, *P* = 0.007) was strongly associated with CR-non-hvKP (Table 1).

**Table 1:** Microbiological and clinical characteristics of CR-hvKP strains.

**Characteristic**	**CR-hvKP** **(*n* = 51)**	**CR-non-hvKP** **(*n* = 29)**	***P*-value**
**Microbiological characteristics**			
Capsular serotype			
K64	44 (86.3)	18 (62.1)	**0.013** ^**b**^
K47	1 (2.0)	1 (3.4)	0.684
K54	1 (2.0)	0 (0.0)	0.451
K-non-typable	5 (9.8)	10 (34.5)	**0.007** ^**b**^
MLST			
ST11	45 (88.2)	20 (69.0)	**0.034** ^**b**^
ST15	5 (9.8)	8 (27.6)	**0.039** ^**b**^
ST198	1 (2.0)	0 (0.0)	0.451
ST438	0 (0.0)	1 (3.4)	0.185
ST-capsular serotype			
ST11-K64	44 (86.3)	17 (58.6)	**0.005** ^**b**^
ST11-K47	1 (2.0)	1 (3.4)	0.684
ST11- K-non-typable	0 (0.0)	2 (6.9)	0.256
ST15-K54	1 (2.0)	0 (0.0)	0.451
ST15- K-non-typable	4 (7.8)	8 (27.6)	**0.017** ^**b**^
ST198- K-non-typable	1 (2.0)	0 (0.0)	0.451
ST438-K64	0 (0.0)	1 (3.4)	0.185
**Clinical characteristics**			
Basic demographics			
Age^a^, years	85.0 (75.0-88.0)	78.0 (69.5-86.0)	0.085
Male	40 (78.4)	20 (69.0)	0.347
Underlying diseases			
Pulmonary disease	47 (92.2)	28 (96.6)	0.435
Diabetes mellitus	26 (51.0)	16 (55.2)	0.718
Hypertension	30 (58.8)	17 (58.6)	0.986
Cerebrovascular disease	25 (49.0)	16 (55.2)	0.597
Cardiovascular disease	31 (60.8)	5 (17.2)	**<0.001** ^**b**^
Malignancy	4 (7.8)	7 (24.1)	0.087
Hypoproteinemia	10 (19.6)	6 (20.7)	0.907
Liver abscess	4 (7.8)	1 (3.4)	0.648
Invasive procedures and devices			
Surgery within 1 month	4 (7.8)	6 (20.7)	0.097
Mechanical ventilation	41 (80.4)	23 (79.3)	0.907
Central venous catheter	35 (68.6)	25 (86.2)	0.081
Urinary catheter	42 (82.4)	27 (93.1)	0.907
Gastric tube	41 (80.4)	28 (96.6)	**0.045** ^**b**^
Antibiotic exposure			
Cephalomycin	7 (13.7)	5 (17.2)	0.674
Penicillin	7 (13.7)	6 (20.7)	0.420
Cephalosporins	27 (52.9)	16 (55.2)	0.847
β-lactam-β-lactamase inhibitors	33 (64.7)	17 (58.6)	0.589
Fluoroquinolones	28 (54.9)	18 (62.1)	0.533
Carbapenems	33 (64.7)	22 (75.9)	0.301
Tigecycline	27 (52.9)	13 (44.8)	0.485
Polymyxin B	3 (5.9)	1 (3.4)	0.633
Glycopeptides	12 (23.5)	2 (6.9)	0.060
Aminoglycosides	6 (11.8)	4 (13.8)	0.793
Fosfomycin	4 (7.8)	3 (10.3)	0.705
Hospitalization within last 90 days	22 (43.1)	7 (24.1)	0.089
Outcomes			
Clinical improvement without modification of initial treatment	8 (15.7)	4 (13.8)	0.821
Change of initial antibiotics due to clinical worsening	30 (58.8)	10 (34.5)	**0.036** ^**b**^
Bacterial clearance after 72 h treatment	2 (3.9)	2 (6.9)	0.560
Persistent infection after 72 h treatment	40 (78.4)	17 (58.6)	0.060
Stable, discharged	25 (49.0)	14 (48.3)	0.949
In-hospital mortality	21 (41.2)	15 (51.7)	0.362
Length of stay^a^, days	24 (15–47)	22 (16.5–34.5)	0.417

*Data are reported using frequencies and percentages if not stated otherwise*.

a *Age and length of stay are presented as mean and standard deviation (SD)*.

b *Bold font means p < 0.05*.

*CR-hvKP, carbapenem-resistant hypervirulent Klebsiella pneumoniae; MLST, multilocus sequence type; ST, Sequence Type*.

### Susceptibility Results and Carbapenemase Resistance Genes

The antimicrobial susceptibility profiles of the CR-hvKP and CR-non-hvKP strains were shown in Table 2 and Supplementary Table 2. All strains were resistant to imipenem and meropenem, except one CR-hvKP strain that is susceptible to meropenem and that showed high resistance to cephalosporins, monobactam, β-lactams-βlactamase inhibitor combinations, and fluoroquinolones but less resistance to ceftazidime-avibactam, imipenem-avibactam, polymyxin B, and tigecycline. Cceftazidime and imipenem MIC_90_ values were significantly reduced by more than sixteen-fold against KPC-2-producing *K. pneumoniae* by combining avibactam (4 μg/mL). There was no statistical significance in the resistance rates of antimicrobial agents between the two subgroups (*p* ≥ 0.05), except polymyxin B (*P* = 0.002). The MIC_50_ values of the following antibiotics of CR- hvKP strains increased two-fold compared with CR-non-hvKP strains: ceftazidime (>128 vs. 128 μg/mL), ceftazidime-avibactam (4/4 vs. 2/4 μg/mL), imipenem-avibactam (0.5/4 vs. 0.25/4 μg/mL), tigecycline (2 vs. 1 μg/mL), levofloxacin (64 vs. 32 μg/mL), cefoperazone-sulbactam (>256/128 vs. 256/128 μg/mL).

**Table 2:** Susceptibility of CR-hvKP and CR-non-hvKP strains against different antimicrobial agents.

**Antibiotic agents**	**CR-hvKP (*****n*** **= 51)**			**CR-non-hvKP (*****n*** **= 29)**			
	**MIC_**50**_ (μg/mL)**	**MIC_**90**_ (μg/mL)**	**Resistance (%)**	**MIC_**50**_ (μg/mL)**	**MIC_**90**_ (μg/mL)**	**Resistance (%)**	***P*-value**
Ceftazidime	>128	>128	100.0	128	>128	100.0	/
Ceftazidime-avibactam	4/4	8/4	2.0	2/4	8/4	3.5	0.684
Cefepime	>128	>128	96.1	>128	>128	100.0	0.283
Aztreonam	>128	>128	100.0	>128	>128	100.0	/
Piperacillin-Tazobactam	>512/4	>512/4	100.0	>512/4	>512/4	100.0	/
Imipenem	>32	>32	100.0	>32	>32	100.0	/
Meropenem	>32	>32	98.0	>32	>32	100.0	0.451
Imipenem-avibactam^a^	0.5/4	2/4	3.9	0.25/4	$\frac{1}{4}$	3.5	0.915
Amikacin	>512	>512	86.3	>512	>512	58.6	0.050
Polymyxin B^b^	0.5	1	0.0	0.5	16	17.2	**0.002** ^**c**^
Tigecycline	2	2	0.0	1	2	0.0	/
Levofloxacin	64	>64	100.0	32	64	100.0	/
Cefoperazone-Sulbactam^a^	256/128	>256/128	100.0	>256/128	>256/128	100.0	/

a *Breakpoints for imipenem-avibactam and cefoperazone-sulbactam have not yet been determined, therefore breakpoints for imipenem and cefoperazone have been applied*.

b *Colistin MIC results can predict the sensitivity of polymyxin B, therefore breakpoints for Colistin have been applied*.

c *Bold font means p < 0.05*.

According to the phenotypic experiment and detection of carbapenemase genes results, all strains in this study showed the presence of KPC-lactamas and carried *bla*_KPC−2_ carbapenemase gene, excluding one CR-non-hvKP strain that produced MBL enzyme and harbored *bla*_IMP−4_.

### MLST Genotyping

The MLST analysis revealed that Sequence type (ST)11 and ST15 were the most common STs in CRKP strains, while the remaining two isolates belonged to ST198 (2.0%, 1/51) and ST438 (3.4%, 1/29). The frequency of ST11 type (88.2 vs. 69.0%, *P* = 0.034) identified in CR-hvKP strains was significantly higher than CR-non-hvKP strains. On the contrary, the proportion of ST15 (9.8 vs. 27.6%, *P* = 0.039) strains was lower (Table 1). The strains were divided into several clones by a combination of capsular genotypes and STs. We found that ST11-K64 (86.3 vs.58.6%, *P* = 0.005) was strongly associated with CR-hvKP, while ST15- K-non-typable (7.8 vs. 27.6%, *P* = 0.017) was correlated with CR-non-hvKP (Table 1).

### Clinical Factors for CR-hvKP Infection of Older Patients

The demographic and clinical factors of patients with CR-hvKP and CR-non-hvKP infections are summarized in Table 1. The age, gender, antibiotic exposure, hospitalization within the last 90 days, and length of stay from CRKP isolation to outcome (in-hospital mortality or discharge) did not have significant differences between CR-hvKP and CR-non-hvKP groups (*P* > 0.05). In addition, no significant differences were observed among most of the underlying conditions such as pulmonary disease, diabetes mellitus, hypertension, cerebrovascular disease, malignancy, hypoproteinemia, and liver abscess (*P* > 0.05), except cardiovascular disease (60.8 vs. 17.2%, *P* < 0.001). Gastric tube was more prevalent in the CR-non-hvKP group than in the CR-hvKP group (96.6 vs. 80.4%, *P* = 0.045), whereas the other invasive procedures and devices had no significant differences. Most of the outcomes between the two groups (*P* > 0.05) had no statistical differences, and patients with a history of change of initial antibiotics due to clinical worsening were more likely to have a CR-hvKP infection than a CR-non-hvKP infection (58.8 vs. 34.5%, *P* = 0.036) (Table 1).

### Risk Factors of CR-hvKP Infection for Older Patients

Nine variables with *P* < 0.05 in univariate analysis were included in the multivariate analysis, including K64, K-non-typable, ST11, ST15, ST11-K64, ST15-K-non-typable, cardiovascular disease, gastric tube, and change of initial antibiotics due to clinical worsening. The results showed that ST11-K64 [odds ratio (OR) = 8.385; *P* = 0.003] and cardiovascular disease (OR = 11.956; *P* < 0.001) were independent predictors of CR-hvKP infections in older patients (Table 3).

**Table 3:** Univariate and multivariate logistic regression analysis of CR-hvKP infections.

**Variables**	**Univariate analysis**		**Multivariable analysis**	
	**OR (95% CI)**	***P-*value**	**OR (95% CI)**	***P*-value**
ST11-K64	4.437 (1.496–13.161)	**0.005** ^**a**^	8.385 (2.017-34.858)	**0.003** ^**a**^
Cardiovascular disease	7.440 (2.438–22.700)	**<0.001** ^**a**^	11.956 (3.072-46.538)	**<0.001** ^**a**^

a *Bold font means p < 0.05*.

*ST, Sequence type; OR, Odds Ratio*.

## Discussions

More than 30 years ago, hvKP was first reported in China (22). In recent years, reports on CR-hvKP have increased gradually, which can spread readily in clinical settings, causing fatal outbreaks (23), and can pose a greater challenge to clinical treatment due to its simultaneous possession of high drug resistance and enhanced virulence (5). According to the China Antimicrobial Resistance Surveillance System report (24), the CRKP strains were more likely to be isolated from older patients, and ICU ranked the top popular unit. Older patients hospitalized in ICU are more prone to nosocomial infections due to low immune function and cumulative basic diseases. In this study, we focused on older patients hospitalized in ICU. Antimicrobial resistance and molecular and clinical characteristics of CR-hvKP strains were reported, showing that the major CR-hvKP clone KPC-2-producing ST11-K64 might have widely disseminated among older patients in ICU.

So far, hvKP is considered to become the predominant cause of pyogenic liver abscess over the past three decades (25, 26). However, the main specimen type of CR-hvKP strains isolated was sputum, and only 7.8% (4/51) patients with CR-hvKP infections had symptoms of liver abscess in our study. Regardless, an important conclusion from this study is that hvKP is not only the main pathogen of suppurative liver abscess, but it is also associated with primary extrahepatic infections, including bacteremia, pneumonia, and soft tissue infections (27, 28).

The production of carbapenemases is the most common mechanism of carbapenems resistant in CRKP strains (29). In the present study, most of the CRKP strains carried *bla*_KPC−2_(*n* = 79) and only one CR-non-hvKP strain harbored *bla*_IMP−4_. As reported in previous studies (30, 31), these CRKP strains were uniformly resistant to ceftazidime, aztreonam, piperacillin-tazobactam, imipenem, levofloxacin, and cefoperazone-sulbactam. Avibactam, as the most effective β-lactamase inhibitor (32), significantly reinstated the efficacy of ceftazidime and imipenem against KPC-producing *K. pneumoniae in vitro*, which decreased the MIC_90_ values of ceftazidime and imipenem by more than sixteen-fold. Unfortunately, in our study, one CR-hvKP strain exhibiting resistant to ceftazidime-avibactam with a MIC value of 16 μg/mL was obtained from patient without previous ceftazidime-avibactam treatment history, which might be due to OmpK35/36 defects, *bla*_KPC−2_ point mutation or higher bla_*KPC*−2_ copy numbers, and gene expressions (33, 34). Whereas the other CR-non-hvKP strain harbored *bla*_IMP−4_ was resistant to ceftazidime-avibactam due to the fact that avibactam is inactive against MBL producing strains (33, 34). Alarming, we found that CR-hvKP strains exhibited high-level MIC_50_ values against six tested antimicrobials compared with CR-non-hvKP strains, including ceftazidime, ceftazidime-avibactam, imipenem-avibactam, tigecycline, levofloxacin, and cefoperazone-sulbactam, respectively. Even though the differences in categorical interpretations for drug resistance between the two subgroups were not striking, elevated MICs might be a critical consideration in clinical management of select cases where the concentration of these toxic antibiotics matters because of underlying comorbidities. However, the results suggested that three “salvage treatments,” ceftazidime -avibactam, polymyxin B, and tigecycline, which were considered to be the most effective treatments for clinical infection caused by carbapenem resistant gram-negative bacteria in contemporary times, still remained potent *in vitro* activities against CR-hvKP, with MIC_50_ and MIC_90_ values 4/4 and 8/4, 0.5 and 1, 2, and 2 μg/mL for ceftazidime-avibactam, polymyxin B, and tigecycline, respectively.

In terms of the thick polysaccharide capsules encoded by *cps* (1), unlike carbapenem-sensitive hypervirulent *K. pneumoniae* that mostly belonged to serotypes K1, K2, or K57 (35), 86.3% (44/51) CR-hvKP strains belonged to capsular genotype K64 in our study (36). By combining capsular genotypes and STs, we found that the ST11-K64 clone was an independent predictor factor for CR-hvKP infection. We speculated that KPC-2-producing ST11-K64 CR-hvKP subclone has been already spreading in ICU. Phylogenetic reconstruction demonstrated that ST11-K64 is evolved from an ST11-K47-like ancestor through recombination and progressively replaced ST11-K47 (37, 38). The ST11-K64 had a remarkably higher 30-day mortality (37), had a significantly higher sepsis/septic shock incidence (38), and had obtained enhanced environmental survival (37). The emergence of KPC-producing ST11-K64 CR-hvKP might pose a serious challenge to the management of older patients infected with these strains in the future. Hence, underscoring the urgent need for timely identification would be important for patient care, and stricter infection control measures should be strengthened to curb the dissemination of these strains in nosocomial settings.

In order to take appropriate intervention measures correctly, a better understanding of risk factors is essential. The univariate analysis showed that the change of initial antibiotics due to clinical worsening was strongly associated with CR-hvKP infection. This reminds that the individualized therapy must be used to treat CR-hvKP infections based on *in vitro* antimicrobial susceptibility profiles, molecular type, and the health status of the patient (39). The cardiovascular disease was an independent predictor of CR-hvKP infections; such patients with underlying diseases typically received inappropriate empiric therapy. However, more frequent hospital contact may result in exposure to CR-hvKP and are more likely to develop hvKP infections (40, 41). While old age and serious complications could reduce immunity and also increase the risk of infection/settlement and even death (42). clinicians should pay close attention to these risk factors in clinical practice to reduce the emergence of CR-hvKP strains.

However, there were some limitations in the present study. First, this is a 1-year retrospective study conducted in a single center, not a multicenter, longitudinal molecular epidemiological study on CR-hvKP, and the number of patients was relatively small. Second, we chose five virulence genes, *rmpA, rmpA2, iucA, iroN*, and *peg344*, which strongly predict hypervirulent phenotype. To preferably determine the hvKP strain, as well as the association between the numbers of virulence-associated genes harbored and virulence of the CR-hvKP strains, *in vitro* and *in vivo* experiments, such as galleria model, mouse model, and human neutrophil experiment, will be performed to evaluate the virulence in the near future. Additional prospective and multicenter studies are needed to confirm these findings in older population.

## Conclusions

In China, CR-hvKP is emerging as a common pathogen in older patients. Cardiovascular disease and ST11-K64 appeared to be independent variables associated with CR-hvKP infections. To our best knowledge, this is the first study in which the CR-hvKP strains showed elevated MIC_50_ values for ceftazidime-avibactam and tigecycline than CR-non-hvKP strains. Higher MICs of the last line antibiotic agents might be a critical consideration in the clinical management of older patients where the concentration of these toxic antibiotics matters because of underlying comorbidities. Caution regarding KPC-2-producing ST11-K64 CR-hvKP as being new significant “superbugs” is required as they are widespread, and infection control measures should be strengthened to curb the dissemination in China.

## Data Availability Statement

The original contributions presented in the study are included in the article/Supplementary Material, further inquiries can be directed to the corresponding author.

## Ethics Statement

The study was approved by the research Ethics Committee of Yueyang Hospital of Integrated Traditional Chinese and Western Medicine, Shanghai University of Traditional Chinese Medicine. Informed consent was not needed due to retrospective nature of the study.

## Author Contributions

HW conceived and designed the study and revised the draft. TW, CZ, and JQ performed the experiments described in this study. JT, LY, and JW collected the laboratory and clinical data. HD, FS, YZ, and HW performed a critical data review. TW performed the statistical analysis and wrote the draft. All authors read and approved the final version of the manuscript.

## Funding

This work was financially supported by the Foundation of Shanghai Municipal Health Commission for Distinguished Young Scholar project (Grant Number: 20204Y0147), the Excellent Youth Incubator Fund Foundation of Yueyang Hospital of Integrated Traditional Chinese and Western Medicine, Shanghai University of Traditional Chinese Medicine (Grant Number: 2019YYQ30), and Chinese Medicine Scientific Research project of Shanghai Municipal Health Commission (2020 JZ001).

## Conflict of Interest

The authors declare that the research was conducted in the absence of any commercial or financial relationships that could be construed as a potential conflict of interest. The reviewer X-YF declared a shared affiliation with one of the authors FS to the handling editor at the time of review.

## Publisher's Note

All claims expressed in this article are solely those of the authors and do not necessarily represent those of their affiliated organizations, or those of the publisher, the editors and the reviewers. Any product that may be evaluated in this article, or claim that may be made by its manufacturer, is not guaranteed or endorsed by the publisher.

## Abbreviations

AST: antimicrobials susceptibility testing
BLAST: Basic Local Alignment Search Tool
cKP: classic *K. pneumoniae*
CLSI: Clinical and Laboratory Standards Institute
cps: capsular serotype-specific
CRKP: Carbapenem-resistant *Klebsiella pneumoniae*
CR-hvKP: carbapenem-resistant hypervirulent *Klebsiella pneumoniae*
CR-non-hvKP: carbapenem-resistant non-hypervirulent *Klebsiella pneumoniae*
PBA: Phenyloronic acid
EDTA: ethylenediamine tetra-acetic acid
*E. coli*: *Escherichia coli*
EUCAST: European Committee on Antimicrobial Susceptibility Testing
FDA: Food and Drug Administration
hvKP: hypervirulent *Klebsiella pneumoniae*
*K. pneumoniae*: *Klebsiella pneumoniae*
ICU: intensive care unit
KPC: *Klebsiella pneumoniae* carbapenemase
MBL: metal β-lactamase
MIC: Minimal Inhibitory Concentration
MLST: Multilocus sequence type
NCBI: National Center for Biotechnology Information
OR: odds ratio
PCR: Polymerase Chain Reaction
SPSS: Statistical Package for the Social Sciences
ST: Sequence type.
